# Prognostic role of high B7-H4 expression in patients with solid tumors: a meta-analysis

**DOI:** 10.18632/oncotarget.8598

**Published:** 2016-04-05

**Authors:** Xing Song, Yingjie Shao, Wendong Gu, Chao Xu, Huihui Mao, Honglei Pei, Jingting Jiang

**Affiliations:** ^1^ Department of Radiation Oncology, The Third Affiliated Hospital of Soochow University, Changzhou 213003, People's Republic of China; ^2^ Department of Laboratory, The Third Affiliated Hospital of Soochow University, Changzhou 213003, People's Republic of China; ^3^ Department of Tumor Biological Treatment, The Third Affiliated Hospital of Soochow University, Changzhou 213003, People's Republic of China

**Keywords:** B7-H4, prognosis, solid tumor, biomarker, meta-analysis

## Abstract

**Background:**

Recently, many studies have shown that B7-H4 exhibits altered expression in various cancers. We performed a meta-analysis to evaluate the prognostic role of B7-H4 expression in solid tumors.

**Results:**

Data from 18 observational studies and 2467 patients were summarized. An elevated baseline B7-H4 was significantly associated with worse OS (pooled HR = 1.79; 95% CI = 1.56–2.06). Differences across subgroups of tumor type, patients' ethnicity, analysis type, HR obtain method and cut-off value were not significant (*P*_D_ = 0.313, *P*_D_ = 0.716, *P*_D_ = 0.896, *P*_D_ = 0.290 and *P*_D_ = 0.153, respectively). Furthermore, patients with high B7-H4 had a significantly shorter DFS (pooled HR = 2.12; 95%CI = 1.45–3.09).

**Materials and Methods:**

We searched PubMed, Embase and the Cochrane Library (last update by November 26, 2015) to identify studies assessing the effect of B7-H4 on survival of cancer patients. Pooled hazard ratios (HRs) for overall survival (OS) and disease-free survival (DFS) were estimated using fixed-effects models and random-effects models respectively.

**Conclusions:**

This meta-analysis clarified that high B7-H4 expression in tissue was significantly associated with poor survival in patients with solid tumors. Future clinical studies are warranted to determine whether B7-H4 blockade has a favorable effect on disease recurrence and mortality.

## INTRODUCTION

Cancer is a leading cause of mortality in the world, and has been a major public health challenge. Based on the GLOBOCAN 2012 estimates, there were approximately 14.1 million new cancer cases and 8.2 million cancer-related deaths in worldwide [1]. Besides early diagnosis, correct therapy strategies based on the prediction of patients' outcome also contributes to successful treatment of cancer. Therefore, it is of great significance for us to identify newer tumor biomarkers with improved sensitivity and specificity to determine the optimal therapeutic strategies and predict the prognosis of cancers. Recently, the costimulatory molecule B7-H4 seems to be a new prognostic marker for cancer.

B7-H4, also known as B7x or B7S1 with the official gene name VCN1 (V-set domain containing T cell activation inhibitor 1) is a member of the B7-family. Proteins in this family are present on the surface of antigen-presenting cells and interact with ligands bound to receptors on the surface of T cells [2]. B7-H4 is a transmembrane protein and belongs to the same family as the inhibitory checkpoint molecule PD-L1. Since a few years, inhibitory checkpoint molecules have been increasingly considered as new targets for cancer immunotherapies. Currently there are more than 6 antibodies against PD-L1 or its ligand PD-1 in late stage clinical trials and two drugs are already approved and in clinical use for lung cancer and melanoma [3].

Compared with PD-L1, the expression of B7-H4 in human cancers is more extensive [4]. Several recent studies have shown that B7-H4 is frequently overexpressed in malignant tumors, including cancers of ovary [5], breast [6], and lung [7]. The expression of B7-H4 in the tumor microenvironment can inhibit the proliferation and activity of T cells, and down-regulate the secretion of immune cytokines such as IL-2 [8, 9]. Knockdown of B7-H4 mRNA and protein expression in the SKBR3 breast cancer cell line enhanced intracellular caspase activity, leading to acceleration of tumor cell apoptosis [10]. Therefore, B7-H4 plays an important role in inducing immunosuppressive effects and may serve as a new target for cancer immunotherapy.

The findings of several clinical studies have suggested that high B7-H4 expression is associated with shorter overall survival (OS) and disease-free survival (DFS) in various types of cancer [11-13]. Nevertheless, the reliability and degree of the prognostic impact of B7-H4 in solid tumors has not yet been methodically analyzed. Therefore, we conducted a systematic review and meta-analysis to assess the prognostic effect of elevated B7-H4 in solid tumors. We hypothesized that high B7-H4 expression represents a biomarker of poor survival in patients with solid tumors.

## RESULTS

### Study characteristics

**Figure 1:**
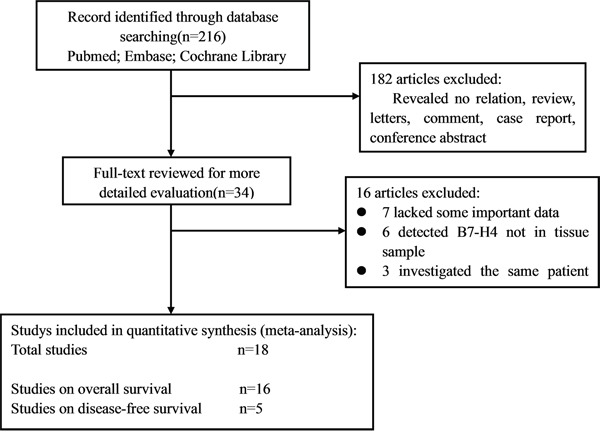
Flow diagram of the study selection process

Using the described searching strategy, 216 references were initially retrieved. After screening the titles, abstracts, publication types and full text of each publication, 34 articles investigated the correlation between B7-H4 expression and patient survival or disease recurrence in various malignant tumors were selected for the systemic review (Table 1). Among these, 16 articles were excluded (seven lacked some important data, six detected B7-H4 not in tissue sample, three investigated the same patient cohorts with others). Finally, 18 studies were enrolled into the meta-analysis (Figure 1) [11-28]. Table 1 shows the main characteristics of the included studies. A total of 2467 patients from China, Japan, Korea, Italy, America, Germany and Greece were diagnosed with a variety of cancers, including esophageal cancer, gastric cancer, colorectal cancer, melanoma, cervical cancer, non-small cell lung cancer, osteosarcoma, ovarian cancer, pancreatic cancer, prostate cancer, thyroid cancer, urothelial cell cancer and so on. Eleven studies (61%) were published in 2013 or later. Fourteen studies (78%) reported on Asians, and 4 studies (26%) on Caucasians. The endpoints OS and DFS were addressed in 16 and 5 studies, respectively. HRs were reported directly in 9 studies and estimated indirectly in the other 9 studies. The cut-off values were different in these studies.

**Table 1:** Main characteristics of all studies included in the meta-analysis

First author [References]	Year	Country	Cancer	Case number	Tumor stage (I/II/III/IV)	Follow-up (months)	Highexpression n (%)	Detected method	Cut-off value	Multivariate analysis	HRs provided from	Outcome measures
**LIU[11]**	2014	Japan	Cervical	102	73/29(I-IIA/IIB-TV)	4.8-169	71 (69.6%)	IHC	NR	yes	Report	OS/DFS
**Liang[12]**	2014	China	Colorectal	185	66/119(I–II/III–IV)	Over60	117(63.2%)	IHC	IRS≥4	yes	Report	OS/DFS
**Chen[16]**	2011	China	ESC	112	13/63/20/16	Longest 111	66(58.9%)	IHC	H-score>160	yes	Report	OS
**Wang[27]**	2015	China	ESC	66	43/23(I–II/III–IV)	Over60	48(72.7%)	IHC	IRS≥4	no	SC	OS
**Arigami[15]**	2011	Japan	Gastric	120	62/58(I–II/III–IV)	Median 40	31(25.8%)	IHC	Moderate staining	yes	Report	OS
**Jiang[13]**	2010	China	Gastric	156	14/22/102/18	Over60	70(44.9%)	IHC	IRS≥9	yes	SC	OS/DFS
**Geng[26]**	2015	China	Gastric	100	40/60(I–II/III–IV)	Over60	71(71%)	IHC	IRS≥3	yes	Report	OS
**Maskey[24]**	2014	China	Gastric	56	12/44(I–II/III–IV)	12-52	12 (21.4%)	IHC	IRS≥9	no	SC	OS
**Quandt[18]**	2011	Germany	Melanoma	29	26/3(III/IV)	Over60	21(72.4%)	IHC	IRS≥9	no	SC	OS
**Li[19]**	2013	China	NSCLC	49	13/29/7(I/II/III)	Over60	20 (40.8%)	IHC	IRS≥9	yes	SC	OS
**Dong[25]**	2015	China	Osteosarcoma	104	62/42(I–II/III)	Over60	73(70.19%)	IHC	IRS≥4	yes	Report	OS
**Simon[14]**	2007	Italy	Ovarian	233	NR	Over60	141(60.5%)	ELISA	protein>426 pg/mg	no	SC	OS
**Chen[22]**	2014	China	Pancreatic	63	NR	1-33	31(49.2%)	IHC	>30% of cells stained	no	SC	OS
**Tsiaousidou[20]**	2013	Greece	Pancreatic	41	4/35/2(I/II/III)	2-31	16(39.0%)	IHC	>10% of cells stained	yes	Report	OS
**Zang[28]**	2007	America	Prostate	823	NR	Median 84	120(15%)	IHC	IRS ≥3	no	Report	DFS
**Zhu[21]**	2013	China	Thyroid	64	30/34(I/II,III,IV)	1-50	46 (71.9%)	IHC	IRS >6	no	SC	OS
**Fan[23]**	2014	China	UCC	62	11/51(Superficial/Invasive)	Over60	47(75.8%)	IHC	IRS≥4	yes	Report	OS
**Jung[17]**	2011	Korea	RCC	102	NR	6-84	18(17.6%)	IHC	>10% of cells stained	yes	SC	DFS

ESC: esophageal squamous cell; NSCLC = non-small cell lung cancer; UCC: urothelial cell cancer; RCC:renal cell cancer; ELISA: enzyme-linked immunoabsorbent assay; IHC: immunohistochemistry; OS = overall survival; DFS = disease-free survival; NR: not report; SC: survival curve; IRS: immunoreactivity score.

### Quality assessment

Each of the 18 eligible studies included in our meta-analysis was assessed for quality according to the Newcastle-Ottawa Quality Assess-ment Scale (NOS). The quality of all studies included varied from 4 to 9, with a mean of 6.3. A higher value indicated better methodology. Therefore, all studies were included in the subsequent analysis.

### Overall survival

Sixteen studies, including 1542 patients, provided suitable data for OS analysis. The main results of this meta-analysis are listed in Table 2. As the studies evaluating OS were not of obvious statistical heterogeneity (*I*^2^ = 0.0%, *P* = 0.481), we used a fixed-effects model to pool the HRs. Overall, the pooled analysis demonstrated that a high B7-H4was statistically significant associated with worse OS (pooled HR = 1.79; 95% CI = 1.56–2.06; *P* < 0.001). A forest plot of study-specific HRs for OS is presented in Figure 2.

**Table 2:** Pooled hazard ratios for OS according to subgroup analyses

Outcome subgroup	No. of patients	No. of studies	Fixed-effects model			Heterogeneity	
			HR (95% CI)	*P* value	*P*_D_ value	*I*^2^ (%)	*P*
Overall survival	1542	16	1.79(1.56,2.06)	<0.001		0	0.481
Ethnicity					0.716		
Asian	1239	13	1.81(1.55,2.12)	<0.001		0	0.560
Caucasian	303	3	1.70(1.24,2.33)	0.001		47.7	0.148
Tumor type					0.313		
ESC	178	2	1.96(1.28,3.02)	0.002		0	0.510
Gastric	432	4	1.74(1.36,2.21)	<0.001		0	0.490
Pancreatic	104	2	2.40(1.37,4.21)	0.002		0	0.702
others	828	8	1.73(1.41,2.11)	<0.001		31.3	0.178
Analysis type					0.896		
Multivariate	1031	10	1.78(1.51,2.09)	<0.001		10.3	0.348
Univariate	511	6	1.82(1.38,2.39)	<0.001		0	0.481
HR obtain method					0.290		
Reported in text	826	8	1.68(1.41,2.02)	<0.001		11.0	0.344
Data extrapolated	716	8	1.97(1.57,2.46)	<0.001		0	0.481
Cut-off value					0.153		
IRS≥4	417	4	1.70(1.31,2.21)	<0.001		21.5	0.281
IRS≥9	290	4	2.45(1.73,3.46)	<0.001		0	0.831
others	835	8	1.67(1.39,2.02)	<0.001		0	0.524

OS = overall survival; HR = hazard ratio; CI = confidence interval; *P*_D_ = *P* for subgroup difference; IRS: immunoreactivity score.

**Figure 3:**
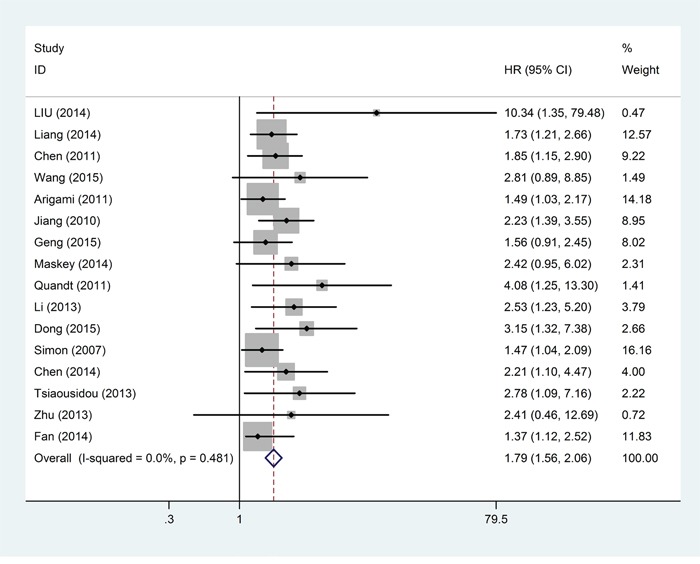
Forest plots of studies evaluating hazard ratios of high B7-H4 expression in solid cancers for overall survival

Pooled HRs for OS according to different cancer sites are shown in Figure 3. The negative effect of elevated B7-H4 on OS was demonstrated in patients with esophageal squamous cell cancer (pooled HR = 1.96; 95% CI = 1.28–3.02; *P* = 0.002), gastric cancer (pooled HR = 1.74; 95% CI = 1.36–2.21; *P* < 0.001), pancreatic cancer (pooled HR = 2.40; 95% CI = 1.37–4.21; *P* = 0.002) and other cancers (pooled HR = 1.73; 95% CI = 1.41–2.11; *P* < 0.001). Differences between tumor type subgroups were not statistically significant (*P*_D_ for subgroup difference = 0.313). B7-H4 was significantly associated with worse OS in Asian patients (pooled HR =1.81; 95% CI = 1.55–2.12; *P* < 0.001) and Caucasian patients (pooled HR = 1.70; 95% CI = 1.24–2.33; *P* = 0.011). Differences across subgroups of patients' ethnicity were not statistically significant (*P*_D_ = 0.716). Also, differences across subgroups of analysis type, HR obtain method and cut-off value were of no significance (*P*_D_ = 0.896, *P*_D_ = 0.290 and *P*_D_ = 0.153, respectively). For OS, statistically significant pooled HR values >1 were consistently calculated in subgroup meta-analyses stratified by patients' ethnicity, tumor type, analysis type, HR obtain method and cut-off value (Table 2).

**Figure 3:**
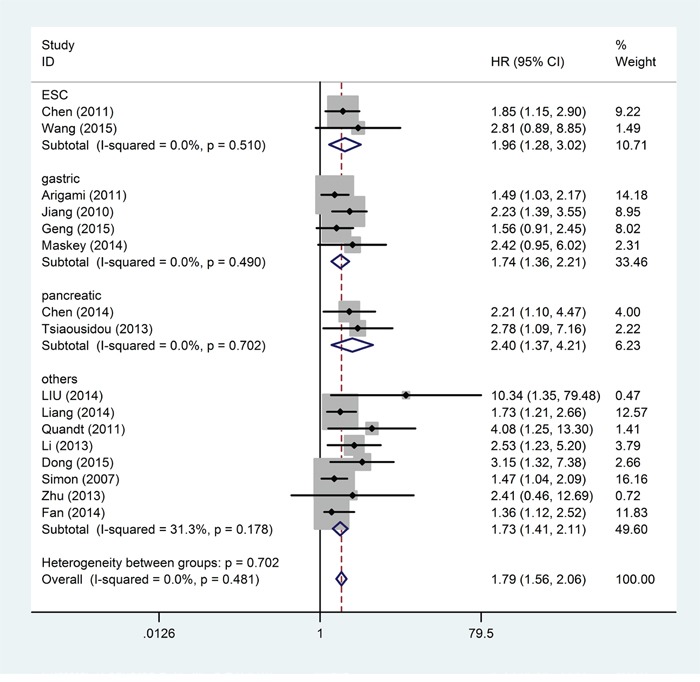
Forest plot of the relationship between high B7-H4 expression and overall survival in patients with a variety of cancers

**Figure 4:**
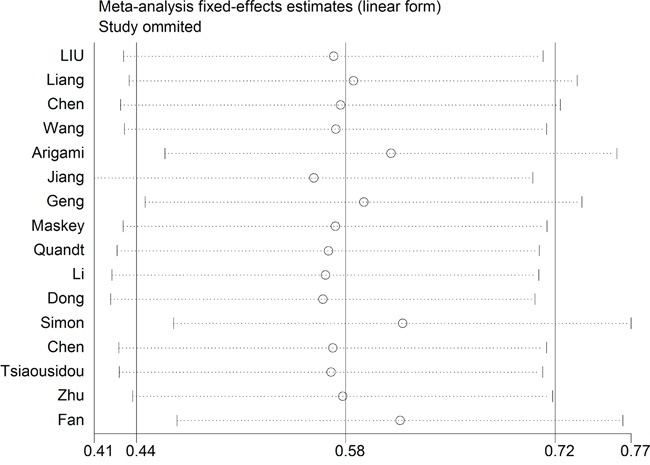
Sensitivity analysis on the relationships between B7-H4 expression and overall survival in solid cancer patients

Sensitivity analysis was performed by sequential omission of individual studies using the fixed-effects model, and the result pattern was not obviously impacted by any single study (Figure 4). We also conducted a meta-regression to explore the potential factors responsible for the heterogeneity. The results showed no statistically significant impact of ethnicity (*P* = 0.998), tumor type (*P* = 0.920), analysis type (*P* = 0.998), HR obtain method (*P* = 0.621) and cut-off value (*P* = 1.000) on the combined effect size for OS.

**Figure 5:**
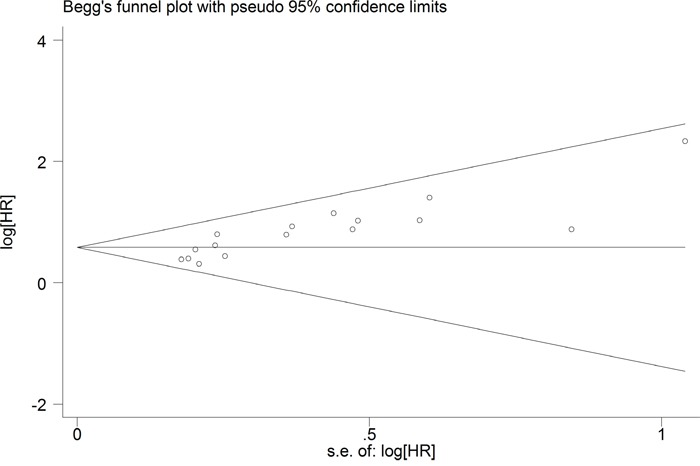
Funnel plots of publication biases on the relationships between B7-H4 expression and overall survival in solid cancer patients

The publication bias of all enrolled studies was evaluated using funnel plots, and Egger's and Begg's tests. Visual inspection of the funnel plot (Figure 5) revealed evidence of publication bias, which was confirmed by Egger's tests (*P* < 0.001). Using the “Trim and Fill” method to adjust for publication bias under the random-effects model, the corrected pooled multivariable-adjusted HR for OS was 1.65 (95% CI = 1.44–1.88).

### Disease-free survival

**Figure 6:**
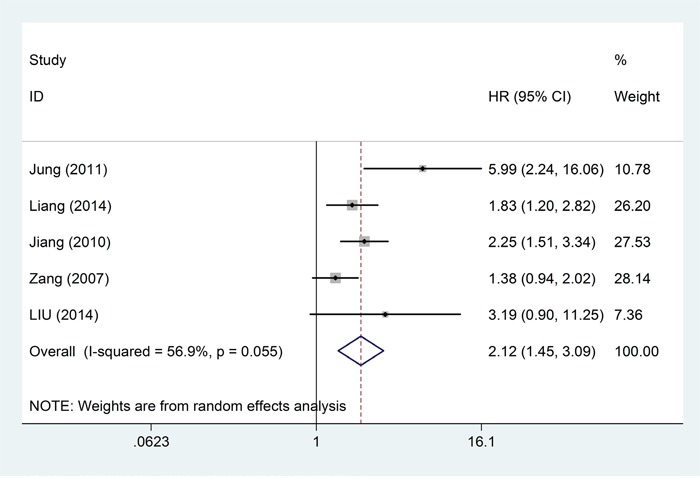
Forest plots of studies evaluating hazard ratios of high B7-H4 expression in solid cancers for disease-free survival

Five studies, comprising a total of 1368 patients, provided suitable data for DFS analysis. Because the five studies that reported DFS were of obvious statistical heterogeneity (*I*^2^ = 56.9%; *P* = 0.055) (Table 3), they were analyzed via a random-effects model and the pooled HR revealed a significant positive association between high level of B7-H4 and poor DFS (pooled HR = 2.12; 95%CI = 1.45–3.09; *P* < 0.001) (Table 3; Figure 6). All the HRs and corresponding 95% CIs are shown in Table 3. A forest plot of study-specific HRs for DFS is presented in Figure 3. Subgroup analysis by cancer site revealed the highest adverse effect of elevated B7-H4 on DFS in patients with renal cell carcinoma (pooled HR = 5.99; 95% CI = 2.24–16.04; *P* < 0.001) (Table 3). A separate meta-analysis of 4 studies (comprising 545 patients) based on Asian patients computed a pooled HR for DFS of 2.44 (95% CI = 1.64–3.65; *P* < 0.001) with moderate heterogeneity among studies (*I*^2^ = 39.8%; *P* = 0.173).

**Table 3:** Pooled hazard ratios for DFS according to patients' ethnicity

Outcome subgroup	No. of patients	No. of studies	Random-effects model			Heterogeneity	
			HR (95% CI)	*P* value	*P*_D_ value	*I*^2^ (%)	*P*
Overall survival	1368	5	2.12(1.45,3.09)	<0.001		56.9	0.055
Ethnicity					0.038		
Asian	545	4	2.44(1.64,3.65)	<0.001		39.8	0.173
Caucasian	823	1	1.38(0.94,2.02)	0.099		-	-
Tumor type					0.055		
RCC	102	1	5.99(2.24,16.06)	<0.001		-	-
colorectal	185	1	1.83(1.20,2.82)	0.006		-	-
gastric	156	1	2.25(1.51,3.34)	<0.001		-	-
prostate	823	1	1.38(0.94,2.02)	0.099		-	-
cervical	102	1	3.19(0.90,11.25)	0.071		-	-

DFS = disease-free survival; HR = hazard ratio; CI = confidence interval; *P*_D_ = *P* for subgroup difference.

## DISCUSSION

The costimulatory molecule B7-H4 is a member of the inhibitory B7 family. High B7-H4 expression has been reported to be involved in tumor immune escape [29]. The functions of B7-H4 in immune escape are achieved mainly through the following three ways: (1) B7-H4-Ig fusion protein could activate regulatory T cells (Tregs), and then inhibit the proliferation and cytokine production of CD4^+^ and CD8^+^ T cells [30]; (2) B7-H4 could reduce the T-cell-stimulating capacity of macrophages, and then suppress tumor-associated antigen-specific T-cell immunity [31]; (3) B7-H4 could arrest cell cycle progression of cytotoxic T lymphocytes (CTLs) in G0/G1 phase [32]. Therefore, the overexpression of B7-H4 in the tumor microenvironment plays an important role in promoting tumor progression and metastasis.

PD-L1, another important inhibitory checkpoint molecule of B7-family, has become a new target for current cancer immunotherapy [33]. PD-L1 can bind with PD-1, and produce inhibitory signals which can inhibit the cytotoxicity of CD8^+^ tumor-infiltrating lymphocytes (TILs) [33]. Compared with B7-H4, PD-L1 has a very different expression pattern. PD-L1 is expressed on some normal tissues, whereas B7-H4 is hardly detected in most non-tumor tissues [34]. PD-L1 is expressed in some human cancers, whereas B7-H4 is highly expressed in numerous types of human cancers, including melanoma [18], breast [6], non-small cell lung [7], ovary [35], kidney [36], prostate [28], esophagus [16], pancreas [37], and stomach cancer [13]. A new study indicated that B7-H1 expression was only correlated with tumor size, whereas B7-H4 expression was correlated with tumor size, lymph-node metastasis, and invasion depth in patients with pancreatic cancer [22]. Statistical analysis showed that high B7-H1 expression were not correlated with patients' overall survival time (*P*=0.089), whereas high B7-H4 expression was correlated with poor survival in pancreatic cancer (*P*<0.001) [22]. Therefore, compared with PD-L1, the expression of B7-H4 in human cancers has higher sensitivity and specificity [4]. These findings suggest that B7-H4 could be a new target for future cancer immunotherapy.

Our meta-analysis provides strong evidence that an elevated B7-H4 is an independent predictor of worse OS in patients with solid tumors. The subgroup analyses showed that the adverse prognostic effect of high B7-H4 remained substantial in different tumor type, as well as for patients of different ethnic backgrounds. Furthermore, this meta-analysis suggests that cancer patients with elevated baseline B7-H4 have a significantly poorer DFS. In addition of B7-H4, B7-H4 mRNA may also be correlated with prognosis in patients with cancer. Although B7-H4 protein expression is absent in most somatic tissues, B7-H4 mRNA has been found to be widely expressed in human peripheral tissues and in most stromal and hematopoietic cells [29, 32, 38]. In tumor tissues, the protein levels of B7-H4 were positively correlated with their mRNA expression. By assessing the relative mRNA expression levels of B7-H4 in tumor tissues and the paired adjacent normal tissues with real-time PCR assay, researchers found that B7-H4 mRNA expression was confirmed in all of tumor specimens and B7-H4 mRNA expression level was higher in tumor tissues than adjacent normal tissues (*P*<0.01) [21, 27]. It was shown that B7-H4 mRNA expression level could also serve as a novel prognostic marker like B7-H4. However, few studies have actually confirmed the correlation between the B7-H4 mRNA and prognosis of cancer patients.

To our knowledge, this is the first meta-analysis providing robust evidence that elevated B7-H4 significantly correlates with worse OS and DFS in patients with solid tumors. However, some details need to be further refined. First, this study included only 18 eligible studies, which resulted in relatively insufficiency data in the subgroup analyses. Second, due to the lack of a unified cut-off value in B7-H4 expression, different cut-off values were used in those studies. The inaccurate cut-off values may affect the availability of B7-H4 as a predictive biomarker in cancer prognosis. In view of this situation, a unified measuring method and cut-off value need to be established. Results of the 18 studies enrolled, 17 measured B7-H4 by immunohistochemistry (IHC), only 1 used enzyme-linked immune absorbent assay (ELISA). Compared with other measuring methods, IHC is more economic and easier to be spread. Thus it can be seen that IHC might be the most common method to measure the B7-H4 expression in the future. Because we found that differences across subgroup of cut-off value were not significant, further studies with larger sample size are still needed to identify the most appropriate cut-off value. Third, several HRs were calculated from the data extracted from the survival curves, which inevitably brought about small statistical errors. Fourth, under common settings, univariate analyses may overestimate effect sizes compared to multivariate analyses. However, univariate analyses based on unadjusted HRs did not show a notable difference in the pooled estimate compared to the multivariate analyses in our study. Finally, funnel plot graphics indicated publication bias. However, we found the corrected pooled effect size remained statistically significant after using the “Trim and Fill” method to adjust the publication bias, and thereby confirming the reliability of our results.

Our results clearly suggest that B7-H4 information could be used to substantially improve prognosis estimation and treatment decision-making of solid tumors. Considering the limitation of present analysis, this conclusion should be regarded cautiously. Further prospective multicentre studies designed adequately with larger sample size are needed to confirm the prognosis value of B7-H4 in cancer patients, as well as to explore more effective therapy strategies.

## MATERIALS AND METHODS

This meta-analysis was carried out in accordance with the Systematic Reviews and Meta-Analyses (PRISMA) guidelines.

### Search strategy

Literatures were searched through PubMed, Embase and the Cochrane Library (last update by November 26, 2015). Keywords used in the search strategy were “B7-H4 OR B7x OR B7S1” (all fields) AND “tumor OR tumour OR neoplasm OR cancer OR carcinoma” (all fields) AND “prognosis OR prognostic OR survival OR outcome” (all fields). We did not impose any advanced limitations when searching the databases. The reference lists of identified articles were also screened to further identify potential studies. The comprehensive database search was carried out independently by two authors (X. Song and Y. Shao).

### Inclusion and exclusion criteria

Literatures that were eligible for inclusion in this meta-analysis met the following criteria: (1) the expression of B7-H4 in cancer tissue; (2) investigation of the relationship between B7-H4 expression level and survival outcome; (3) provided sufficient data to estimate the hazard ratio (HR) and 95% confidence intervals (CI). Considering the tumorigenesis and metastasis mechanism of hematological malignancy is different from other tumors of epithelial origin, studies of hematological malignancy were excluded. When multiple studies reported on the same patient cohort, only the most recent or complete study was selected. Case reports, letters, reviews, conference abstracts and animal trials were excluded. Two reviewers independently evaluated titles and abstracts of the identified articles and subsequently excluded those that were considered irrelevant. The full text of enrolled articles was carefully examined for comprehensive evaluation. Disagreement from two reviews was resolved by consensus.

### Data extraction and quality assessment

The required information from all eligible studies was collected by two researchers independently, which included first author's surname, publication year, origin of population, tumor type, sample number, tumor stage, lymph node metastasis, follow-up period, detected source, detected methods, the cut-off value, and HR as well as corresponding 95% CI. If a study reported both the results of univariate and multivariate analysis, only the latter was selected because it has increased precision due to accounting for confounding factors.

The quality of each study was assessed independently by two researchers according to the NOS [39]. For quality assessment, scores ranged from 0 (lowest) to 9 (highest), and studies with scores of 6 or more were rated as high quality.

### Statistical analysis

High and low expression of B7-H4 was defined according to the cut-off values provided in the articles. HRs and their 95% CIs were combined to measure the effective value of elevated B7-H4 expression on prognosis. If the statistical variables were described in the study, we extracted them directly. Otherwise, they were calculated from available numerical data in the articles according to the methods described by Tierney [40]. The data from Kaplan-Meier survival curves were read by Engauge Digitizer version 4.1, and three independent researchers read the curves to reduce reading variability. We also sent e-mail to the corresponding authors of eligible articles requesting additional information and original data needed for the meta-analysis. An observed HR greater than 1 indicated a worse prognosis in patients with B7-H4 overexpression and HR less than 1 suggested a better prognosis. Statistical heterogeneity was assessed by visual inspection of forest plots, by performing the Chi-square test (assessing the *P* value), and by calculating the *I*^2^ statistic [41, 42]. If the *P* value was less than 0.05 and/or *I*^2^ exceeded 50%, indicating the presence of heterogeneity, a random-effects model (the DerSimonian-Laird method) was used. Otherwise, the fixed-effects model (the Mante-Haenszel method) was used. Subgroup analysis and meta-regression were further performed to explore the source of identified heterogeneity. Publication bias was estimated by visually assessing the asymmetry of an inverted funnel plot. Furthermore, Begg's and Egger's tests were performed to provide quantitative evidence of publication bias. If publication bias was observed, we adjusted for the effect by the use of the Duval and Tweedie trim-and-fill method [43]. For all analyses, STATA version 12.0 (Stata Corporation, College Station, TX, USA) was used with significance defined as a *P*-value of less than 0.05 except where otherwise specified.
